# Consumer Expectations of Online Services in the Insurance Industry: An Exploratory Study of Drivers and Outcomes

**DOI:** 10.3389/fpsyg.2017.01254

**Published:** 2017-07-27

**Authors:** M. Dolores Méndez-Aparicio, Alicia Izquierdo-Yusta, Ana I. Jiménez-Zarco

**Affiliations:** ^1^Business Administration, University of Burgos Burgos, Spain; ^2^Faculty of Economics and Business, Open University of Catalonia Barcelona, Spain

**Keywords:** expectations, reputation, perceived usefulness, subjective norm, prior recommendation

## Abstract

*Today, the customer-brand relationship is fundamental to* a company’s bottom line, especially in the service sector and with services offered via online channels. In order to maximize its effects, organizations need (1) to know which factors influence the formation of an individual’s service expectations in an online environment; and (2) to establish the influence of these expectations on customers’ likelihood of recommending a service before they have even used it. In accordance with the TAM model (Davis, 1989; Davis et al., 1992), the TRA model (Fishbein and Ajzen, 1975), the extended UTAUT model (Venkatesh et al., 2012), and the approach described by Alloza (2011), this work proposes a theoretical model of the antecedents and consequences of consumer expectations of online services. In order to validate the proposed theoretical model, a sample of individual insurance company customers was analyzed. The results showed, first, the importance of customers’ expectations with regard to the intention to recommend the “private area” of the company’s website to other customers prior to using it themselves. They also revealed the importance to expectations of the antecedents perceived usefulness, ease of use, frequency of use, reputation, and subjective norm.

## Introduction

Since the start of the current decade, an extensive body of literature has been published on the factors influencing the formation of customer service expectations, in both online and offline environments. This issue is especially important for companies such as insurance companies, which base their innovation strategies on the use of the Internet as a channel for customer relations and dialog (PWC, 2017).

Obviously, what a consumer expects to receive upon using a service is influenced by his or her perception of the brand behind it, as well as by that brand’s ability to deliver on its promises (Lassoued and Hobbs, 2015). Likewise, the pressure exerted by a subject’s immediate environment influences service expectations (De Leeuw et al., 2015; Bilgihan et al., 2016). Thus, third-party opinions influence the outcomes individuals expect to receive after consuming or using a brand, but they can also affect consumers’ feelings about the brand, such as brand love, even in the absence of experience-based antecedents (Roy et al., 2013)

Tornatzky and Fleischer’s (1990) methodological proposal added another factor to the model-building process. In addition to the aforementioned factors, these authors held that it was essential to consider other factors inherent to the technology used to support the service offering. This is because the characteristics that subjects perceive of the technological tools used to deliver a service exert a considerable influence on the expected outcome in terms of both: (1) the company’s ability to meet their needs (Venkatesh et al., 2003), and (2) the relationship the subject establishes with it (Wang et al., 2016).

Because of the instrumental nature of technology, the research conducted in this field has focused on how technology influences the process of purchasing, obtaining, and using a service (Thakur and Srivastava, 2014). In this regard, Yucel and Gulbahar (2013) and McKechnie et al. (2006) found that the degree to which individuals perceive that using a given technology will make it easier to obtain a service and the degree to which they believe it will be useful for obtaining certain benefits are key factors in the formation of individual expectations.

Analyzing the antecedents of individual expectations is essential to understanding what leads a person to recommend the use of a company’s service (López and Sicilia, 2013). From a marketing perspective, recommendation is vital to future success (Hui et al., 2007), as it exerts a considerable influence on the behavior of prospective customers. According to Nielsen (2015), in 92% of purchases, consumers base their decision on the recommendation they receive from friends and acquaintances as opposed to other sources. This is especially important in the online context, where WOM has become one of the most reliable and credible sources (Chari et al., 2016). As noted by Wang and Benbasat (2007), the potential user assumes that the person recommending the brand is trustworthy and familiar with it. The recommendation thus enhances the brand’s credibility and new users become more likely to try the product or service (Hsu et al., 2013; Lu et al., 2014; Hanssens et al., 2015).

**FIGURE 1:**
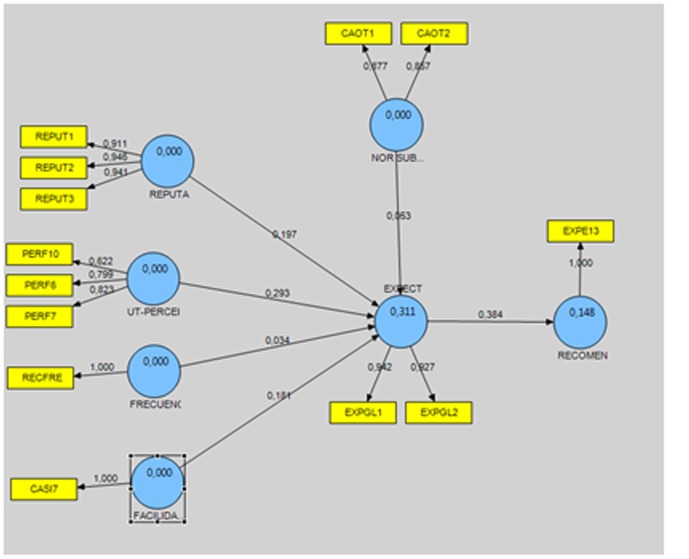
Structural model.

In this line, a StrongView (2014) report by StrongView and Edison Research revealed two key figures: (1) 77% of people make their purchases based on the recommendation received; and (2) people recommend a brand when it greatly exceeds their expectations. Ladhari et al. (2011) and Meleddu et al. (2015), among others, have argued that recommendation is a behavior typical of the post-purchase and use stage and that the individuals who engage in it are the people with high levels of brand satisfaction, loyalty, and engagement. However, sometimes the recommendation is made early on in the purchasing process, i.e., in the pre-purchase stage (Roy et al., 2013; Ruiz et al., 2014). In this stage, the individual does not yet have experience using or consuming the service; therefore, the only driver for recommendation would seem to be the existence of high and positive expectations for the outcome (Roy et al., 2013).

Knowing what elements influence consumers’ expectations, as well as the implications – or consequences – thereof as concerns recommendation, is key in strategic terms for service companies. Among service companies, the insurance industry in particular should be given special attention, due to the important role that insurance plays in the economy by enabling the assumption of risks and mobilizing savings. When it works well, it contributes to economic growth and financial stability. With assets worth two thirds of EU GDP, the EU insurance industry is a significant player in the financial sector. In some countries, such as Spain, the industry accounts for as much as 5.5% of GDP (González and Marques, 2014).

In the insurance industry, knowing the antecedents and consequences of expectations will: (1) make it possible to tailor the service offering to customers’ needs; (2) increase credibility and boost consumers’ trust in the company; (3) increase customer engagement; and (4) facilitate the attraction of new customers. According to a recent PWC (2017) report, today digitalization is a reality for insurance companies. The challenge, however, is not only to increase digital revenue, but also to consolidate the adoption and dissemination of the Web channel among customers (Nicoletti, 2016). Expectation formation clearly plays a fundamental role in this process, both for customers trying the Web channel for the first time and for those who already have experience using it. The intangibility, inseparability, and heterogeneity of services lead even customers with use experience to have different expectations with regard to the outcome (Qureshi and Bhatt, 2015).

Given the virtual non-existence of literature on this line of research, and drawing on the above ideas, the present paper proposes a theoretical model of the antecedents of individual online insurance service expectations, as well as their consequences in terms of pre-purchase recommendation. To this end, the remainder of this paper is organized as follows. First, a theoretical framework is developed and the hypotheses to be tested are proposed. Next, the fieldwork and results are described. Finally, the implications for business are discussed.

## Theoretical Framework and Hypotheses

### Individual Expectations

The literature offers various definitions of this concept, following two main proposals, those of Oliver (1980) and Parasuraman et al. (1988). In the early 1980s, Oliver defined the expectation-disconfirmation paradigm, stating that “expectations are consumer-defined probabilities of the occurrence of positive and negative events if the consumer engages in some behavior” (Oliver, 1981). In contrast, the gap-based service-quality model developed by Parasuraman et al. (1988) defines expectations in terms of what customers feel they should be offered. The former has been restated as “predictive expectations” and the latter as “desired expectations” (Yi, 1990).

Today, conceptualizations of expectations generally fall within one or the other line of thinking. However, the lines share certain features: (1) they recognize that expectations are the result of an individual, and (2) they entail awaiting a given result. In this regard, Zeithaml et al. (1993) found that consumer expectations are pretrial beliefs about a given product that serve as a standard or reference point against which to judge its performance. Spreng et al. (1996) defined them as “beliefs about a product’s attributes or performance at some time in the future.” In contrast, for Wu et al. (2014) they are “generalized beliefs that individuals have about a social object.” Finally, Kujala et al. (2017) have noted that expectations determine what an individual expects to receive from a service and that they, in turn, are conditioned by the desires and level of abstraction the individual achieves during the evaluation process.

In the online environment, the above definitions are fully valid. However, in addition to considering the psychological nature of expectations, the influence of technology on the quality, performance, or experiences individuals receive both while consuming the service and during the purchasing process must also be considered (De Keyser and Lariviere, 2014).

Traditionally, expectation research in the field of services has consistently referred to quality, since customers’ main objective is for the service they intend to purchase to meet certain quality standards (Parasuraman et al., 1988; Alén and Rodríguez, 2004). With online services, technology is key to quality; therefore, characteristics such as ease of use, the quantity and timeliness of the information provided, or the speed, precision and security with which it enables completion of the process, among others, have all been identified as dimensions of service quality (Zavareh et al., 2012). However, expectations may also be related to how or what an individual might feel when consuming the service (Koenig-Lewis and Palmer, 2014). While many of these feelings and emotions develop from the purchasing and consumption process itself, others are the result of the characteristics of the environment in which these processes take place (Nakamura and Csikszentmihalyi, 2014).

In this regard, technology once again plays an essential role, since it can generate flow experiences in subjects that leave them both deeply involved in an enjoyable activity and emotionally absorbed (Ozkara et al., 2016). However, although technology influences the expectation formation process, it is the individual who ultimately carries the process out. Hence, internal factors related to how individuals perceive the brand, their experience with it, their personal characteristics, and the third-party recommendations they receive directly influence the expected future service outcome (Oliver, 1977, 1980; Cadotte et al., 1987; Oliver and Burke, 1999; Andreassen, 2000; Torres Moraga, 2010; Duque-Oliva and Mercado-Barboza, 2011; all cited in Pelegrín-Borondo et al., 2016).

### Characteristics of the Technology: Service Adoption Models

Understanding what drives an individual to use technology has been one of the chief concerns among management scholars and professionals. A great deal of the literature has focused on comparing the predictive capacity of the different theories on technology adoption and use. Many of these studies, such as Gounaris and Koritos’s (2008) study on the adoption of online banking, have shown that incorporating concepts from various theoretical frameworks increases a model’s explanatory power.

Most of the theoretical developments have been based on the Theory of Reasoned Action (TRA), formulated by Ajzen and Fishbein between 1975 and 1980. This theory holds that individual behavior depends on intentions, which, in turn, depend on attitude and social pressure (subjective norm) to engage in the behavior (Montano and Kasprzyk, 2015). Thus, any other factor that might influence behavior does so only indirectly. In the 1980s, the TRA was widely used. Its great explanatory power and consistency with other studies made it suitable to predict a broad set of behaviors (Davis et al., 1989). Indeed, such was the TRA model’s importance that it was the basis for two subsequent lines of work: the Theory of Planned Behavior (TPB) and the Technology Acceptance Model (TAM). Both lines corroborate the role of individual intentions as triggers of individual behavior.

Sampedro et al. (2014) have argued that the TPB offers a general model that explains individual conduct based on the beliefs-attitude-intention-behavior relationship. The intention to act is considered the best indicator of the behavior, since it is indicative of the effort the individual is willing to make to perform a given action. The model is completed with the inclusion of three exogenous variables that explain the behavioral intention: attitude toward the behavior, subjective norm, and perceived control in the behavior.

Meanwhile, the TAM is perhaps the model to receive the most attention in academia. Developed by Davis (1989) and Davis et al. (1992), the TAM is an adaptation of the TRA model that focuses on the behavior of new technology use.

Analytically simplifying the previous models, the TAM places special emphasis on analyzing the factors affecting individual attitudes and intentions. Hence, it proposes that the decision to use a technology is based on its degree of functionality and the characteristics of the interface (Yucel and Gulbahar, 2013). In particular, the TAM predicts that the use of ICT is conditioned by two specific individual beliefs about technology: (1) perceived usefulness (PU), and (2) perceived ease of use (PEOU).

Perceived usefulness was first introduced as a factor in the TAM model. Subsequently, Venkatesh et al. (2003) included it as part of the concept of performance expectancy. Davis (1989) defined it as “the degree to which a person believes that using a particular system would enhance his or her job performance.” Individuals thus generate this perception by assessing whether a new system offering additional features would enhance their performance compared to that achieved with a system previously used to carry out the same function or even before using any system at all. Perceived usefulness can thus be considered extrinsic to the technology itself, related instead to efficiency and effectiveness in the performance of an activity and to expectation formation.

*H1. The perceived usefulness of a technological application positively influences a user’s service outcome expectations.*

Perceived ease of use is also part of the TAM and refers to “the degree to which a person believes that using a particular system would be free of effort” (Davis, 1989). This concept was introduced in the Innovation Diffusion Theory (IDT), but in the opposite sense, in terms of complexity (Rogers, 1962; Moore and Benbasat, 1996). Subsequently, Venkatesh et al. (2003) conceptualized the idea as the construct *effort expectancy*.

This factor is relevant to the adoption of a system, because systems that are easy to use require a smaller effort on the part of the user, thereby allowing him or her to allocate more resources to other activities, since effort is a finite resource (Radner and Rothschild, 1975). In this regard, Davis (1989) holds that a system that is easy to use will generate a more positive attitude in the user toward using it. Furthermore, like perceived usefulness, PEOU is subjective in nature and, thus, can vary from one individual to the next in relation to the same system. The perception will be different depending on the user’s knowledge or prior experience with similar systems. Finally, PEOU can also vary over time. As users become more proficient in the use of a system, they may begin to perceive it as increasingly easy to use (Martins et al., 2014). In light of these considerations, the following hypothesis was formulated:

*H2. The PEOU of a technological application has a dual effect on the user’s service outcome expectations.*

In general, the TAM is the most widely applied theoretical system in the field of information systems. Because of this widespread use, it is considered a well-established and robust theory (Svendsen et al., 2013; Yucel and Gulbahar, 2013).

However, over time, certain aspects of the TAM, related to its definition, design, and direction of implementation, have proven limited. Baron and Ensley (2006) identified important problems in the model, related to the definition of its key constructs. Similarly, Venkatesh et al. (2003, 2012) and Bagozzi (2007) have highlighted the need to increase the model’s explanatory power by including additional variables and even the need for a paradigm shift in this regard. In other fields, authors such as Goh et al. (2016) have also underscored the need to increase the model’s explanatory power through the inclusion of additional variables.

### Characteristics of the Service User

In addition to the characteristics of the technology used to support the service, online service expectations also depend on the individual, on his or her perception of and relationship with the brand, and on the influence exerted on him or her by the environment.

#### The Influence of External Recommendations

One of the main contributions of the TRA (Fishbein and Ajzen, 1975) lies in its recognition of the influence of social environment on individual behavior. Referring to the subjective norm, these authors asserted that individual behavior is strongly shaped by the influence on the individual of certain important groups or actors.

The most common conceptualization of this phenomenon in the literature on the influence of social factors is that of the subjective norm imposed by reference groups. The subjective norm is an external driver related to the beliefs of others – people whom the individual regards as important – who provide the individual with information that he or she considers credible and relevant and, thus, ultimately strongly influence his or her behavior (Venkatesh et al., 2012). Bagozzi and Dholakia (2002) noted that individuals incorporate the opinions of a referent – an individual or group – that is important to them as part of their own belief structure, making them their own. Thus, third-party opinions affect the attitudes, motivations, and expectations the individual shows with regard to performing a behavior (Rivis and Sheeran, 2003; Hsu and Lu, 2004).

For the subjective norm to be positive, the individual has to perceive that his or her reference group approves of the behavior (Bhatti, 2007). The basis for this norm is twofold: individual motivation and the beliefs of the people the individual regards as referents. The motivation to perform the behavior lies in the individual’s need to be accepted by the influencing group, while beliefs refer to the individual’s conviction that the group’s opinion is the best and most favorable for him or her (Küster and Hernández, 2013).

Other times, the influence is exerted by some other agent or institution, such as the manufacturer, retailer, or brand. With brands, the influence exerted on an individual’s motivations, expectations, and behavior is determined by the credibility this source has for the individual (Westerman et al., 2014). Perceived source credibility has been defined as “judgments made by a perceiver concerning the believability of a communicator” (O’Keefe, 1990). While the precise factor structure of source credibility is still being debated (Cronkhite and Liska, 1976), one of the most common factor structures includes three dimensions: expertise or competence (i.e., the degree to which the perceiver believes the source knows the truth), trustworthiness (i.e., the degree to which the perceiver believes the source can be trusted to tell the truth to the best of his or her knowledge), and goodwill (i.e., the degree to which the perceiver believes the source has the perceiver’s best interests at heart.)

The subjective norm, whether exerted by friends, family members, and peers or by any other agent, plays a decisive role in the performance of certain social behaviors, including purchasing and consumption behavior. Social influence is likewise decisive in the purchase of certain products or brands and in their use in certain contexts or situations (López de Ayala López et al., 2015).

Nevertheless, some studies do not include the social norm as an antecedent of individual expectations and behavior, citing the lack of empirical evidence supporting the relationship between social influence and behavioral intention (Davis, 1989). Mathieson (1991) added that this null influence of the reference groups could be due to methodological aspects, such as the choice of the behavior to be studied, the sample composition, or the scope of the research. Based on the above ideas, the following hypothesis was formulated:

*H3. External recommendations positively influence users’ service outcome expectations.*

#### Influence of Corporate Reputation

The literature offers various definitions of this concept. Alloza et al. (2013) define corporate reputation as the *set of collective evaluations that a company’s behavior evokes in different audiences, motivating their conducts of support or opposition*. Similarly, Gotsi and Wilson (2001) and Schwaiger et al. (2009) argue that reputation is a general evaluation of a company over time. According to these authors, this evaluation is made based on: (a) the stakeholder’s direct experience with the company, or (b) any form of symbolism that provides information about the company’s actions and/or offers a comparison with the actions of its leading rivals.

Despite the diversity of definitions, all share certain common features. The first is that reputation has a temporal dimension, which distinguishes it from image or identity (Cravens and Oliver, 2006). Additionally, reputation arises as a result of interactions between stakeholders and the organization over time (Argenti and Druckenmiller, 2004). Consequently, an organization does not have just one reputation, but rather as many reputations as there are groups with which it interacts. Finally, reputation is the result of the evaluation individuals make regarding the behavior of the organization or brand.

Helm (2007) has noted that there is considerable agreement on the positive effects of having a good reputation. Gotsi and Wilson (2001) highlighted the relationship between reputation and individuals’ perceptions of an organization, although here it must be added that the evaluation made directly influences the individuals’ future expectations of the company or brand’s behavior (Walker, 2010). A good reputation leads to high expectations, as people consider there is no dissonance between what the company promises and what it delivers (Walsh et al., 2016). Fombrun (2011) argued that reputation is the basis for trust. This feeling is of enormous importance, as it is the trigger for favorable attitudes and behaviors toward a brand or organization. A good reputation is based on good work, on fulfilling the promises an organization makes in response to its stakeholders’ expectations. Accordingly, Alloza (2011) has suggested that a good reputation generates confidence, while at the same time increasing stakeholder retention and satisfaction. Roberts and Dowling (2002) indicate that a good reputation generates positive outcomes for an organization, influencing its survival and financial performance. According to Firestein (2006), reputation is the strongest determinant of an organization’s sustainability. While strategies can always be changed, once a reputation has been seriously harmed, it is difficult for an organization to recover. Based on the above ideas, the following hypothesis was formulated:

*H4. Reputation positively influences the user’s service outcome expectations.*

#### Use Experience

Prior experience using and consuming a product has traditionally been considered one of the most important variables moderating the variation in attitudes toward a brand or company (Lee and Ma, 2012). This experience is the result of direct or indirect contact with a company. Direct contact generally occurs during the purchasing process, or during the use of a product or service, and is usually initiated by the customer. In contrast, indirect contact usually consists of unplanned encounters with representations of a company’s products, services, or brands in the form of recommendations or verbal critiques by other customers, advertising, news reports, reviews, etc. As a result, individuals acquire knowledge of the brand and its attributes, leaving them in a position to compare the outcomes obtained with their initial expectations.

In the early 1990s, Johnson and Fornell (1991) and Zeithaml et al. (1993) found that the consumption experience is the most important factor for the formation of future expectations. These expectations then become the basis for evaluating the service outcome and making judgments about satisfaction (Oliver, 1980) and quality (Parasuraman et al., 1991). Additionally, Karapanos (2013) found that prior experience is the most important source provided by a product for acquiring knowledge about the brand.

Experience accumulates over time. The more experience an individual has with a brand or product, the greater his or her degree of familiarity with it becomes. Consequently, the product’s perceived quality is likely to change over time, as is the relative importance of certain qualities. For instance, while learnability and novelty may play a vital role at the start, other aspects, such as usefulness or social capital, may ultimately drive prolonged use (Hassenzahl, 2004). One would thus expect different levels of experience to lead to changes in consumer attitudes. However, the literature reveals numerous contradictions in this regard. For example, the theory of assimilation (Hovland et al., 1957) and the theory of contrast (Singer et al., 1972) offer a different perspective on the effect of expectations on satisfaction. In light of these considerations, the following hypothesis was formulated:

*H5. Prior experience with the service positively influences the user’s service outcome expectations.*

### The Effects of Expectations: Recommendation

One of companies’ main objectives is to get customers to recommend the use of their service. In the marketing area, recommending means suggesting or advising that a certain product, service, or brand is the best in its category to meet a given need (Skålén et al., 2015). Research on marketing has highlighted the key role that expectations play in service recommendation (Parasuraman et al., 1988). Consumers define their expectations based on different service characteristics and outcomes (Oliver, 1980; Alén and Rodríguez, 2004). At the same time, individuals will recommend a product or service: (1) when, based on their use experience, they are able to confirm that the product or service has met their expectations (Gupta and Harris, 2010); or (2) when, despite not having use experience, they have a high degree of trust in the brand or company, and, therefore, in its ability to fulfill its promises with regard to the product or service, based on which they formed their expectations (Esteban et al., 2014).

In the first case, consumers make the recommendation once they have used the service, upon determining that the outcome was satisfactory (Flavian et al., 2014). In other words, once they have used the service, consumers’ own experience allows them to evaluate whether the final outcome exceeds, falls short of, or matches their initial expectations. In online environments, confirmation of customers’ prior expectations positively influences their satisfaction level (Kim, 2012). Thus, prior customers form their expectations based on their perception of certain characteristics of the seller, such as reputation, size, the information available in the media, etc. Customer satisfaction is the result of a post-purchase and post-service-use evaluation and comparison process that affects customers’ intention both to recommend the service to other customers and to re-use the company’s services themselves (i.e., user loyalty) (Yi, 1990; Anderson et al., 1994). Consequently, customers’ willingness to recommend a service is often used in marketing literature to analyze the relationship between customer satisfaction and customer loyalty (Rodríguez del Bosque et al., 2006; Lin and Lekhawipat, 2014).

Notwithstanding the above, with online services, each use situation can be considered a new experience (Blut et al., 2014). The evaluation of the outcome and its comparison with prior expectations are complex. The intangible, inseparable, and heterogeneous nature of services means that each use situation can result in a different outcome. Consequently, even if prior experience with a service influences expectations, there may still be a certain degree of uncertainty with regard to the expected outcome.

According to Chaudhuri and Holbrook (2001), brand loyalty has been defined as a deeply rooted emotional commitment to the brand that leads the customer to consistently engage in certain repetitive behaviors in the future, such as: (a) rebuying or repatronizing a preferred product or service, or (b) recommending it to others. These behaviors result in repeated recommendation or purchasing of the same brand despite situational influences and marketing efforts that could potentially cause the customer to switch to a different brand.

As this description shows, even in the face of negative external influences, the customer still feels compelled to repurchase, recommend, and commit to the brand over others. The idea of brand loyalty assumes: first, the existence of an actor with free will; second, that this actor is focusing his or her free will on an object; and, third, that brand loyalty is built over time (Pearson, 2016). This long-term brand-loyalty relationship can moreover be developed in two different dimensions: a behavioral dimension and an attitudinal one (Söderlund, 2006). Bandyopadhyay and Martell (2007) and Veloutsou (2015) have discussed whether both dimensions are needed to achieve a higher level of brand loyalty, known as “true brand loyalty”.

The behavioral dimension of brand loyalty is identified based on customers’ observable and repetitive behavioral patterns at a given time. Such patterns would include re-purchasing a preferred item or recommending the brand to others. In contrast, the attitudinal dimension consists of customers’ non-observable patterns and is thus built on attitudes, intentions, and the strength of the customer’s relationship with the brand (Dick and Basu, 1994; Bandyopadhyay and Martell, 2007).

The online context has underscored the strategic importance of recommendation. Marketing literature recognizes recommendation as a direct effect of satisfaction, such that satisfaction increases the likelihood that consumers will convey positive information about brands or products. Known as word-of-mouth or WOM, this post-consumption behavior is considered to positively affect both the brand’s reputation and prospective customers’ purchasing decisions (López and Sicilia, 2013). Satisfied customers whose prior expectations are confirmed when they use a company’s online process are more likely to recommend it, making them (the customers) promoters of the company. In an online context, the effect of WOM is further amplified, as a result of the Internet’s global reach (Yoo et al., 2013; López and Sicilia, 2014).

In the second case, despite not having experience using the service (Roy et al., 2013; Esteban et al., 2014), the customer exhibits a high degree of trust in the brand or organization and, thus, in its ability to fulfill its promises regarding the service on which the customer’s expectations are based (Esteban et al., 2014). In other words, the lack of use experience suggests that it is individuals’ brand expectations that lead them to make the recommendation.

Wilson et al. (2017) found that some individuals develop a strong emotional link with the brand, known as “brand love,” which increases the levels of brand loyalty and positive recommendation. Similarly, Esteban et al. (2014) noted that the personal link between consumer and brand has become a strategic issue for companies, as its main consequences include, among other things: brand trust (Albert and Merunka, 2013); willingness to pay a brand premium (Thomson et al., 2005); and willingness to forgive brand failures (Batra et al., 2012).

According to Esteban et al. (2014), brand love can be defined as an intimate and emotional tie between an individual and a brand, characterized by a set of different beliefs (e.g., personal integration with the brand), feelings (emotional connection, affiliation), and behaviors (desire to use it, willingness to recommend it, etc.).

Roy et al. (2013) showed that brand love can develop either based on experience using the brand – or the online service offered – or through managed (firm-sponsored) or unmanaged (word-of-mouth) communication about the brand. The first kind of antecedent is related to a satisfying brand experience over time, while the second is related to the non-experiential antecedent of brand love. In this situation, the origin of this emotional bond lies in the brand’s reputation and the external influence – subjective norm – exerted on the individual by his or her environment. Thus, consumer beliefs about the brand and the services it can offer, as well as such external communication, act as cues for consumers, leading them to develop certain perceptions and attach meaning to brands even when they have no direct experience with them.

Bergkvist and Bech-Larsen (2010) identified two other factors that can promote brand love based on experience using a brand – or online service – namely, consumer-brand identification and the sense of community of the brand’s users. With regard to brand identification, Ahuvia (2005) found that love objects are central to how people identify. More recently, Belk (2013) and Sheth and Solomon (2014) showed that this feeling is also found in the digital world. Similarly, Bagozzi and Dholakia (2006) proposed the concept of brand identification as the “extent to which the consumer sees his or her self-image as overlapping the brand’s image.” Bergkvist and Bech-Larsen (2010) showed that identification means “self-image congruence” and “self-connection” and also noted that previous studies, such as Fournier (1998) and Kressmann et al. (2006), had found a positive relationship between brand identification and brand passion and love.

The desire of be part of a community – sense of community – can also promote love of a brand and its services even among consumers with no experience using it. In this regard, Bagozzi and Dholakia (2006) applied the concept of social identity in the context of brand community. In particular, they noted that social identity is positively related to brand identification, because increased identification with the brand community leads to greater engagement with the brand, which, in turn, leads to an assimilation of the brand’s identity into one’s own. Bergkvist and Bech-Larsen (2010) defined “sense of community” as the kinship or affiliation a consumer feels with other people associated with the brand and showed that sometimes this feeling becomes a need for the individual, thereby strengthening his or her bond with the brand, as well as his or her desire to belong to the community.

All of these elements increase the individual’s expectations with regard to the service’s expected outcome. Similarly, Hegner et al. (2017) found that these factors are conducive to this behavior, insofar as they raise the individual’s outcome expectations in terms of both the Web channel and the service. In light of these considerations, the following hypothesis was proposed:

*H6. The user’s service outcome expectations (positive/negative) influence recommendation (positive/negative) of the service.*

## Research Methodology

In order to ensure homogeneous data and enable the comparison of user/consumer assessments, a specific service was chosen, namely, an insurance company. This choice made it possible to compare the results obtained by variable (e.g., reputation or expectations). To this end, an insurance company was selected that provided coverage throughout Spain and had a high number of insured. The selected insurance company ranks fourth in terms of market share (7.44%) out of a total of 100 insurance companies operating in Spain (Investigación Cooperativa entre Entidades Aseguradoras y Fondos de Pensiones [ICEA], 2016). Of the entire insured population, those individuals listed as registered in the private area for clients were chosen. This condition was predetermined by the research objective. Specifically, the target customers were those customers who had used the company’s online services in the last month (23,223 customers in total). Once the users/customers had been selected, they were e-mailed an invitation to access a questionnaire. Access was voluntary, but as an incentive to complete the questionnaire, they were offered a small reward: a discount coupon for the purchase of fuel. The technical details of the research are shown in **Table 1**.

**Table 1:** Technical details of the research.

Universe	Registered users of the private area of Company XX’s website who had used this service at least once
Sampling procedure	By means of a structured survey accessed online
Sample	23,223
Scope	Spain
Real sample	4,178
Sampling error	±1.05%
Level of confidence	95% (*Z* = 1.96)
Maximum variance allowed	*P* = *q* = 50%
Fieldwork	June/July 2016

Between June 9 and 26, 3 waves of e-mails were sent out to these users with a link to the online survey. A total of 4,178 surveys were completed. To obtain the data, a structured questionnaire was used, with closed, single-response questions. By age group, 30% of the respondents were between the ages of 36 and 45; 25% were between the ages of 26 and 35; 25% were between the ages of 46 and 55; 14% were between the ages of 56 and 65, 4% were over the age of 65; and only 2% were under the age of 25. Of the total number of respondents, 85% had prior experience using the private area for clients; of these, the experience of 81.6% was with this company.

To test the proposed hypotheses, scales used in previous studies were selected based on the literature review. Ten-point Likert scales were used to measure the variables, as they are the most suitable for this type of research. Specifically: (a) Likert scales are the most suitable for measuring individual attitudes and perceptions (Likert, 1932; Krieg, 1999); (b) they are suitable for obtaining information through the design of online surveys (Mathieson and Doane, 2005); and (c) they are suitable for use in causal models (Krieg, 1999). The scales used and the description and codification of the items are shown in Annex I.

The structural model used (**Figure 1**) was validated using the partial least squares (PLS) regression technique. The model was estimated using SmartPLS 2.0 software, and the significance of the parameters was established through bootstrapping with 4,178 subsamples the same size as the original. To ensure convergent validity, all indicators with a factor loading that was not significant or was less than 0.7 were eliminated. The resulting model thus had no reliability problems, according to the established criteria (Cronbach’s alpha, composite reliability, average variance extracted) (**Table 2**). As can be seen in **Table 2**, two of the scales had a Cronbach’s alpha less than 0.7. In this regard, Loewenthal (1996) suggested that a reliability score of 0.6 can be considered acceptable for scales with fewer than 10 items. Likewise, Nunnally (1978), Cronbach and Shavelson (2004), Huh et al. (2006), and Malhotra (2008) have suggested that scores greater than 0.6 can be considered acceptable.

**Table 2:** Reliability and convergent validity of the model.

Factor	Indicator	Mean	Std deviation	Loading	*T*-value	Cronbach’s α	Compound reliability	AVE
REPUTA	REP3	7.86	1.945	0.86	28.70^∗∗∗^	0.92	0.95	0.86
	REP4	6.40	2.882	0.71	40.67^∗∗∗^			
	REP5	7.50	2.118	0.90	36.28^∗∗∗^			
	REP6	7.24	2.198	0.91	34.68^∗∗∗^			
	REP9	7.61	2.063	0.95	27.27^∗∗∗^			
	REP1	7.23	2.071	0.81	41.87^∗∗∗^			
	REP7	7.15	2.158	0.89	38.42^∗∗∗^			
	REP8	7.35	2.001	0.92	35.10^∗∗∗^			
	REP10	7.21	2.081	0.91	36.23^∗∗∗^			
	REP2	7.18	2.018	0.91	36.42^∗∗∗^			
SUBNORM	CAOT1	4.73	3.328	0.677	26.48^∗∗∗^	0.67	0.85	0.75
	CAOT2	3.14	3.029	0.867	24.52^∗∗∗^			
PERUTIL	PERF10	5.89	2.834	0.622	22.93^∗∗∗^	0.61	0.79	0.56
	PERF6	7.81	2.446	0.799	35.03^∗∗∗^			
	PERF7	7.93	2.449	0.823	37.82^∗∗∗^			
EXPECT	EXPEC1	7.01	1.974	0.76	55.12^∗∗∗^	0.85	0.93	0.87
	EXPEC2	7.38	1.848	0.90	69.25^∗∗∗^			
	EXPEC3	7.31	2.219	0.81	60.51^∗∗∗^			
	EXPEC4	7.42	1.939	0.86	65.13^∗∗∗^			
FREQUENC	FREQ	3.15	2.495	NA	NA	NA	NA	NA
PEASYUSE	CASI7	7.70	1.931	NA	NA	NA	NA	NA
INTENTION	INTENT	7.39	2.061	NA	NA	NA	NA	NA

*NA, Not applicable; ^∗∗∗^*p* < 0.01*.

To assess the discriminant validity, the average variance extracted for each factor was used, taking into account that it should be greater than the square of the correlation between each factor pair (Fornell and Larcker, 1981), as indicated in **Table 3**.

**Table 3:** Discriminant validity.

	EXPECT	PEASYUSE	FREQUENC	SUBNORM	INTENTION	REPUTA	PERUTIL
EXPECT	**0.934**						
PEASYUSE	0.4331	**NA**					
FREQUENC	0.0918	0.0410	**NA**				
SUBNORM	0.2189	0.1882	-0.0143	**0.867**			
INTENTION	0.3843	0.4176	0.0534	0.1821	**NA**		
REPUTA	0.4260	0.6045	-0.0356	0.3064	0.6429	**0.932**	
PERUTIL	0.4581	0.4147	0.2001	0.2441	0.2871	0.3554	**0.753**

*Off-diagonal elements are the estimations of the correlations among the factors. The diagonal elements (in bold) are the square root of the average variance extracted*.

Once the measurement instrument’s psychometric properties had been evaluated, the structural model synthesizing the proposed hypotheses (**Figure 1**) was estimated using PLS, and the same criteria were used to determine the significance of the parameters (bootstrapping with 4,178 subsamples, the same size as the original sample) (Efron, 1982; Efron and Gong, 1983; Efron and Tibshirani, 1993; Chin, 1998a,b, p. 320; Streukens and Leroi-Werelds, 2016).

In order to evaluate the structural model’s predictive capacity, the criterion proposed by Falk and Miller (1992) was used, whereby the R^2^ of each dependent construct must be greater than 0.1. Lower values, even if significant, should not be accepted. This made it possible to determine whether there was support for the proposed hypotheses based on the significance of the standardized estimated regression coefficients (**Table 4**).

**Table 4:** Testing of hypotheses.

Hypothesis	Standardized β	Bootstrap *t*-value
H1: Perceived usefulness > Expectations	0.293^∗∗∗^	15.73
H2: Perceived ease of use > Expectations	0.184^∗∗∗^	7.88
H3: Subjective norm > Expectations	0.053^∗∗∗^	4.14
H4: Reputation > Expectations	0.197^∗∗∗^	8.80
H5: Frequency of use > Expectations	0.034^∗∗^	2.47
H6: Expectations > Intention to recommend	0.384^∗∗∗^	22.11

*R^*2*^ Expectations = 0.311; *R*^*2*^ Intention to recommend = 0.148. ^∗∗∗^*p* < 0.001; ^∗∗^*p* < 0.05*.

## Results

The results obtained in the model showed, first, that there was support for all the proposed hypotheses. Second, they showed that the most important effects were the effects of expectations on the intention to recommend using the company’s website to perform transactions and process claims (β = 0.384; *p* < 0.01; H6), followed by the influence of perceived usefulness on expectations (β = 0.293; *p* < 0.01; H1). The effects of reputation and PEOU on expectations came third, with nearly identical values (β = 0.197, *p* < 0.01 for H4 vs. β = 0.184, *p* < 0.01 for H2). Last but not least was the influence of the subjective norm (β = 0.053; *p* < 0.01; H3) and of frequency of use (β = 0.034; *p* < 0.01; H5) on expectation formation.

This research verified that expectations strongly influence a customer’s intention to recommend using the private area for clients to other customers to process their claims. These results were consistent with previous studies, which have highlighted the relationship between expectations, satisfaction, and loyalty (Lin and Lekhawipat, 2014; Kanthachai, 2015), as well as the intention to purchase and/or use a product or service. This finding would also be in keeping with those established by the TPB and TAM models used in other contexts (e.g., online banking or tourism) (Martins et al., 2014). With regard to the drivers of expectations, the results show the importance of the drivers of attitude – perceived usefulness and PEOU – from the TAM (Davis, 1986, 1989) and TRA (subjective norm) (Fishbein and Ajzen, 1975), both of which hold that for a technology to be used, individuals must perceive that the benefit of using it outweighs that which they would obtain if they did not use it. Finally, attention should be drawn to the importance of the variable *reputation* in the formation of expectations, as a mitigator of perceived risk and a quality signal emitted by the company (Kirmani and Rao, 2000).

## Conclusion

The results of this research have partially remedied the lack of previous research (a) using reputation, perceived used, ease of use, subjective norm, and frequency of use as joint antecedents of expectations, and (b) on recommendation to use a private area for clients before the recommending customer has used the feature him or herself. In other words, this paper sought to assess aspects that influence the likelihood of recommendation of an online service prior to the process of purchasing or using it.

One key finding was that one way to ensure use of a private area for clients (available exclusively online and to the users of a service) is to encourage the users/customers by emphasizing the important benefits to be obtained from using it, as proposed by McKechnie et al. (2006) and Yucel and Gulbahar (2013). Another key finding was that, despite the high rate of Internet use and the various omnichannel strategies on the market, customers are reluctant to use the private area of a website for their transactions with a company. Instead, they prefer to perform transactions through the traditional channel, because of the personalized nature of the service, using the rest of the channels for less risky procedures (to check information, look for sales, etc.).

In light of these findings, first, companies should look for ways to encourage the users of the private area to act as vehicles for disseminating the benefits of its use. To this end, they could use strategies based on customizing advertising messages to feature actual customers and creating discussion forums to generate buzz about the benefits (eWOM). WOM is both one of the main information sources used by users and the most reliable (Chari et al., 2016). For instance, a recent Nielsen (2015) study found that 95% of consumer purchase decisions are based on recommendations received from friends and acquaintances as opposed to other sources.

Second, this research has shown that the drivers of the formation of customer expectations are determined by such important variables as perceived usefulness, reputation, subjective norm, and PEOU. The study sought to determine how consumer/user expectations are formed with regard to the use of an online service as opposed to the provision of the same service offline or through traditional channels. In this regard, many of the studies conducted on the TAM have demonstrated the importance of perceived usefulness. The present findings are consistent with previous studies (e.g., Wu and Wang, 2005; Kim et al., 2008; Hess et al., 2014; Izquierdo-Yusta et al., 2015). By way of example, attention should be drawn to the perceived value model proposed by Zeithaml (1998), which served as the inspiration for several subsequent studies highlighting the influence of perceived value on consumers’ behavioral intentions (Dodds et al., 1991; Grewal et al., 1998). From the perspective of the insured, use of the private area of the website should facilitate the entire process, allowing the insured to carry out all processes easily and entailing cost savings (whether in terms of waiting times, travel, opportunity costs, etc.). Therefore, companies should design their websites to be accessible through a small number of clicks, provide fast downloads, be easy to navigate, etc.

Finally, a company’s reputation is more important for companies that do not yet have a firmly established online presence than for those that use multiple channels, since in omnichannel environments, consumers/customers form their expectations based on their experiences in offline environments or on knowledge acquired in traditional channels. In this regard, a strong reputation can positively influence customer attitudes (determined by perceived usefulness and PEOU) toward a company’s products or services or the channels through which it operates (Erdem and Swait, 2004), as well as the formation of their expectations and the subsequent intention to purchase/use the company’s product or service or recommend it. Accordingly, reputation serves as a credible signal that companies send to the market and that consumers use to deal with information asymmetries (Fombrun and Shanley, 1990; Kirmani and Rao, 2000). Consequently, companies should seek to create and/or cultivate or invest in their reputation, as opposed to acting opportunistically (Kirmani and Rao, 2000), making it a source of competitive advantage and, thus, of transaction cost savings.

## Future Lines Of Research And Limitations

This exploratory study enabled the assessment of consumer behavior in online environments. Specifically, it looked at the intention to recommend use of an online service from the perspective of the expectations individuals form prior to using the service themselves. Future lines of research should seek to assess the outcome of this process, i.e., satisfaction, trust, and loyalty. Also, it should be interesting to compare between registered and unregistered users.

The main limitation of the present study lies in the selected sample, i.e., insurance company users. Subsequent research should seek to test the model in another industry with several different products and/or services.

## Author Contributions

All authors listed have made a substantial, direct and intellectual contribution to the work, and approved it for publication.

## Conflict of Interest Statement

The authors declare that the research was conducted in the absence of any commercial or financial relationships that could be construed as a potential conflict of interest.
