# Supplementary material for: Consumer Expectations of Online Services in the Insurance Industry: An Exploratory Study of Drivers and Outcomes
Source: Front Psychol. 2017 Jul 27;8:1254. doi: 10.3389/fpsyg.2017.01254 (PMC5529399; doi:10.3389/fpsyg.2017.01254)
Supplement: Supplementary file 1 [file Data_Sheet_1.docx]

ANNEX I

**VARIABLES, CODIFICATION, DESCRIPTION, MEASUREMENT AND BIBLIOGRAPHICAL REFERENCES**

| **Description of the item** |  | **Measurement** | **Bibliographical references** |
| --- | --- | --- | --- |
| **PERCEIVED EASE OF USE**  It is compatible with the features of my device (ELIMINATED)  It is always available (ELIMINATED)  It is quick (ELIMINATED)  It is easy to use  It has a very attractive design (ELIMINATED) | CASI2  CASI3  CASI4  CASI7  CASI5 | Likert (1-10) | Adapted from Davis (1989),  Davis et al. (1992) |
| **PERCEIVED USEFULNESS**  Because I manage my business online  Because it suits my schedule best  Because it offers me more information  Poor performance of the rest of the channels (ELIMINATED) | PU6  PU7  PU10  PU8 |  | Adapted from Davis (1989),  Davis et al. (1992) |
| **SUBJECTIVE NORM**  Because the company recommended it to me  Because the people around me use it regularly | CAOT1  CAOT2 |  | Adapted from Davis (1989) |
| **EXPECTATIONS**  My expectations were generally high  I expected high-quality information to help me perform my process smoothly  I expected to perform a quality process as if I had used the personalized channel  I expected a website with high-quality technology | EXPEC1  EXPEC2  EXPEC3  EXPEC4 |  | Devlin et al. ( 2002), Knutson et al. (1990), Kuo-Chien Chang et al. (2014),  Swaid and Wigand (2012),  Parasuraman et al. (1991) |
| **REPUTATION**  My insurance at Company XX covers everything I need  My company’s insurance is the best and I do not compare prices with other companies  My company’s staff is highly qualified  My company’s channel offers all the processes and information required to meet my needs  This company is a modern company that naturally applies new technologies  This company has excellent technical and technological resources that allow me to perform all my operations online  This company innovates in its products according to new market needs  I think this company has always protected me and I feel safe and at ease with the company  This company is honest and ethical  In short, I feel this company has an excellent reputation | REP3  REP4  REP1  REP7  REP8  REP10  REP2  REP5  REP6  REP9 |  | Doney and Cannon (1997),  Fombrun et al. (2000), Fombrun and Shanley (1990), Walsh et al. (2009),  Walsh and Beaty (2007) |
| **INTENTION TO RECOMMEND**  Before using the private area service, how likely were you to recommend Company XX to a family member or friend? | INTENT |  | The authors |
| **FREQUENCY OF USE**  I use the private area 1= rarely, 2=once a month, 3= every 15 days, 4= once a week, 5= twice a week | FREQ | Likert (1-5) | The authors |
